# Neuromuscular Diseases and Bone

**DOI:** 10.3389/fendo.2019.00794

**Published:** 2019-11-22

**Authors:** Giovanni Iolascon, Marco Paoletta, Sara Liguori, Claudio Curci, Antimo Moretti

**Affiliations:** Department of Medical and Surgical Specialties and Dentistry, University of Campania “Luigi Vanvitelli”, Naples, Italy

**Keywords:** neuromuscular diseases, osteoporosis, fractures, physical activity, vitamin D, glucocorticoids, rehabilitation

## Abstract

Neuromuscular diseases (NMDs) are inherited or acquired conditions affecting skeletal muscles, motor nerves, or neuromuscular junctions. Most of them are characterized by a progressive damage of muscle fibers with reduced muscle strength, disability, and poor health-related quality of life of affected patients. In this scenario, skeletal health is usually compromised as a consequence of modified bone–muscle cross-talk including biomechanical and bio-humoral issues, resulting in increased risk of bone fragility and fractures. In addition, NMD patients frequently face nutritional issues, including malnutrition due to feeding disorders and swallowing problems that might affect bone health. Moreover, in these patients, low levels of physical activity or immobility are common and might lead to overweight or obesity that can also interfere with bone strength features. Also, vitamin D deficiency could play a critical role both in the pathogenesis and in the clinical scenario of many NMDs, suggesting that its correction could be useful in maintaining or enhancing bone health, especially in the early phases of NMDs. Last but not least, specific disease-modifying drugs, available for some NMDs, are frequently burdened with adverse effects on bone tissue. For example, glucocorticoid therapy, standard of care for many muscular dystrophies, prolongs long-term survival in treated patients; nevertheless, high dose and/or chronic use of these drugs are a common cause of secondary osteoporosis. This review addresses the current state of knowledge about the factors that play a role in determining bone alterations reported in NMDs, how these factors can modify the biological pathways underlying bone health, and which are the available interventions to manage bone involvement in patients affected by NMDs. Considering the complexity of care of these patients, an interdisciplinary and multimodal management strategy based on both pharmacological and non-pharmacological interventions is recommended, particularly targeting musculoskeletal issues that are closely related to functional independence as well as social implications.

## Definitions and Pathophysiological Issues

Neuromuscular disease (NMD) is an umbrella term encompassing different categories of disorders affecting anterior horn cells, peripheral nerves, neuromuscular junctions, and skeletal muscles (Table 1). If we exclude diabetic neuropathy, when considered individually, NMDs are rare diseases, although overall involve a considerable population.

**Table 1:** Neuroanatomical classification of neuromuscular diseases.

**Lesion level**	**Hereditary**	**Acquired**	**Abbreviation**
Anterior horn	Spinal muscular atrophy Amyotrophic lateral sclerosis		SMA ALS
		Post-polio syndrome	PPS
Nerve	Charcot–Marie–Tooth disease	Guillain–Barre syndrome Chronic inflammatory demyelinating polyneuropathy Neuropathy with monoclonal gammopathy of unknown significance	CMT GBS CIDP MGUS
Neuromuscular junction		Myasthenia gravis Lambert–Eaton myasthenic syndrome	MG LEMS
Muscle	Duchenne muscular dystrophy Becker muscular dystrophy Facioscapulohumeral dystrophy Limb–Girdle muscular dystrophies Emery–Dreifuss dystrophy Oculopharyngeal muscular dystrophy Myotonic dystrophy Pompe's disease		DMD BMD FSHD LGMD EDD OPMD DM PD
		Polymyositis Dermatomyositis	PM DM
	Inclusion body myositis		IBM

Even if muscle weakness is a typical finding in several NMDs, a heterogeneous clinical scenario characterizes these conditions involving different organs and systems. Moreover, severe functional implications occur, forcing affected patients to be wheelchair-bound in advanced stages. As a consequence, both the pathology itself and the complex clinical management lead to a huge economic and social impact, also because in most of cases NMDs affect young people.

Although muscle impairment represents the main determinant, bone damage could play a role in the functional prognosis and consequently in the level of quality of life. From both physiological and pathological points of view, muscle and bone are connected by various pathways and mechanisms. The bone–muscle cross-talk includes several biological factors that could affect bone metabolism in NMDs independently of mechanical load.

Muscle tissue releases specific molecules, called myokines, that could interact with bone through different pathways. Myostatin, a transforming growth factor-beta (TGF-β) superfamily member, is a growth and differentiation factor highly expressed in muscle in pathological conditions leading to muscle atrophy. At the same time, myostatin modulates bone metabolism, promoting osteoclast differentiation and inducing bone loss (1).

On the other hand, muscle contraction promotes the release of bone protective molecules, including irisin, which stimulates osteoblasts and inhibits osteoclast activities through the NFkB signaling pathway, increasing cortical bone thickness and reducing the risk of fragility fractures in osteoporotic women (2, 3).

In this scenario, other players have been identified, such as β-aminoisobutyric acid (BAIBA) and musclin, which might improve bone accrual and prevent bone loss by activating osteoblast precursors and by inhibiting osteoclastogenesis (4, 5). Interestingly, the complexity of the biological interaction between muscle and bone is further supported by the evidence that about 50% of patients with inclusion body myositis caused by valosin-containing protein (VCP) gene mutation develop Paget's disease of bone (6).

Indeed, the VCP protein is implicated in membrane trafficking to facilitate the ubiquitin proteasome system in its function to eliminate aberrant proteins, and it also has a role in autophagy-mediated protein aggregation with its dysfunction leading to pathological aggregates in bone, muscle, and brain. These findings suggest a putative genetic susceptibility to bone metabolic disorders in NMDs (6).

Karasik et al. (7) identified three main periods, where this link occurs, including embryonic life, the postnatal period (allometric growth), and adult life (homeostatic relationship). This conceptual model will guide our review addressing the relationship between muscle and bone in the most common NMDs. However, in this review, we cannot include diabetic neuropathy because of the complexity of the pathophysiological mechanisms underneath bone damage in diabetic disease that is beyond the scope of this review.

## Epidemiology

NMDs are rare diseases (prevalence < 1/2000) that require challenging management strategies in terms of diagnostic and therapeutic approaches to address several clinical and functional issues (8).

Although our knowledge and the diagnostic tools in NMDs have been improved during the last decades, the prevalence of these conditions has not significantly increased compared with the past (9). However, myotonic dystrophy (MD) has doubled its prevalence, probably due to the use of more accurate genetic tests. The age distribution of NMDs is quite variable. Some of these diseases have early onset in childhood, such as Duchenne muscular dystrophy (DMD) and congenital muscular dystrophies, while amyotrophic lateral sclerosis (ALS), post-polio syndrome (PPS), Lambert–Eaton myasthenic syndrome (LEMS), and inclusion body myositis (IBM) have an onset in adulthood. However, some NMDs are characterized by a uniform age distribution, namely, Charcot–Marie–Tooth disease (CMT), Guillain–Barré syndrome (GBS), myasthenia gravis (MG), facioscapulohumeral dystrophy (FSHD), and MD. Regarding gender distribution, most NMDs equally affect men and women. Nevertheless, ALS, chronic inflammatory demyelinating polyneuropathy (CIDP), GBS, LEMS, DMD and Becker muscular dystrophies (BMD), and FSHD are more prevalent in males, whereas MG, polymyositis (PM), and dermatomyositis (DM) are more prevalent in females (10).

Although the prevalence of each NMD is very low worldwide, the early onset, the longer survival rates related to new available therapies, and the progressive worsening of the clinical conditions and physical performance impose a heavy social and economic burden. The costs of NMDs include medical expense, costs for the caregiver, and use of medical or external devices such as ventilators, orthoses, and wheelchairs for improving the clinical condition and the quality of life of affected individuals.

Per-patient medical costs for NMDs are more than double the estimated mean all-cause disability-associated healthcare expenditures (11) in particular, the costs are two times higher for DMD (12). The increase in healthcare costs depends not only on cardiorespiratory complications but also on bone damage along with a dramatic rise in costs related to fractures, particularly for patients affected by DMD who lose the ability to ambulate independently (13). The progressive loss of muscle power and strength and of the ability to stand up are associated with poor bone health and increased risk of falls, leading to higher rate of fragility fractures. For instance, in patients with spinal muscular atrophy (SMA), the prevalence of these fractures ranges from 9.3 to 46%, and they commonly occur at the distal femur (14). However, epidemiological data about fragility fractures are widely variable in various NMDs due to different patterns of disease progression. Indeed, in DMD patients, the prevalence of vertebral fractures (VFs), often undiagnosed, is estimated up to 30% commonly occurring in older, non-ambulatory patients, whereas limb fragility fractures (femur or tibia) affect from 20 to 60% of younger, ambulatory patients (15). However, VFs are strictly related to glucocorticoids (GCs) use considering that these drugs mainly affect the trabecular bone.

## NMDs Involving Anterior Horn Cells

SMA is a typical and clarifying example of anterior horn cells disease. This condition is an autosomal recessive disorder due to absence or low level of survival motor neuron protein codified by survival motor neuron genes (SMN 1 and 2) located on long arm chromosome 5. Homozygous deletion of SMN1 is diagnostic of SMA and the clinical variability depends on the SMN2 copy number that defines the level of protein produced (16).

Considering the embryonic period in the bone–muscle cross-talk, SMA genotypes characterized by deletions or point mutations regarding exons 6 and 7 of the SMN genes showed enhanced production of cytokines that stimulate the formation and activation of osteoclasts, resulting in increased bone resorption (17, 18). In the Smn^−/−^SMN2 mouse model, a notable decrease of bone mineral density (BMD) and caudal vertebra alterations along with pelvic fractures compared to wild types were reported. Similarly, prenatal SMA 0 patients showed a phenotype characterized by reduced bone mass mainly due to decreased load on the fetal skeleton through reduction of fetal movement (19). However, also in the postnatal and adult period of bone–muscle cross-talk, skeletal involvement in SMA is strongly correlated to the severity of the muscular impairment [Table 2; (14, 19–22)].

**Table 2:** Classification of clinical variant of SMA.

**Type**	**SNM2 Copy Number**	**Onset**	**Life Survival**	**Motor Milestones**	**Peculiar Findings**	**Bone Involvement**
**SMA 0**	1	Prenatal	Weeks	None achieved	Severe hypotonia Respiratory distress at birth	Generalized osteopenia Bone deformities
**SMA I** (Werdnig–Hoffmann disease)45% of cases	2–3	0–6 months	Death prior to age 2	Sit with support	Bell-shaped deformity of the chest “Frog-leg” lower limb posture Feeding problems (suck and/or swallowing)	Lowest aBMD *Z*-scores at all skeletal sites Higher fracture risk Osteoporosis^*^
**SMA II** 20% of cases	3	<18 months	70% alive at age 25 years	Independent sitting achieved by 9 months or delayed Never stand or walk independently	Postural tremor of fingers (minipolymyoclonus)	Increased bone resorption markers Low aBMD *Z*-scores at the LS and the LDF Fragility vertebral fractures
**SMA III** (Kugelberg–Welander disease)30% of cases	3–4	IIIa 18 months−3 yearsIIIb 3–30 years	Normal	Independent ambulation	No available data	Increased bone resorption markers Low aBMD *Z*-scores at the LDF Asymptomatic vertebral fractures
**SMA IV** Less than 5% of cases	4 or more	Adulthood	Normal	Normal	No available data	No available data

* *According to 2015 ISCD pediatric criteria; aBMD, areal bone mineral density; LS, lumbar spine; LDF, lateral distal femur*.

Wassermann et al. reported low BMD in SMA patients, including one to three subtypes. In particular, patients affected by SMA 1, despite an initial improvement of lateral distal femur (LDF) BMD from 3 to 10 years old, exhibited BMD *Z*-scores declining during adolescence and, however, normal BMD values are never reached. On the contrary, patients affected by SMA 3 show a reduction of LDF-BMD later, as weight bearing declines. Furthermore, significantly increased fracture risk is reported in all subtypes, particularly for SMA 1, typically affecting lower limbs (14). A recent 36-month study investigating bone health in prepubertal SMA type 2 and 3 population showed low serum 25-OH vitamin D3 [25(OH)D], high serum parathyroid hormone (PTH), and bone resorption markers [C-terminal telopeptide (CTx)] with bone formation markers [bone-specific alkaline phosphatase (BSAP)] within normal ranges. Furthermore, low lumbar spine (LS) BMD and increased asymptomatic VFs were reported (21). In particular, Baranello et al. claimed that, in SMA patients, bone loss occurred regardless of muscle wasting, suggesting a multifactorial mechanism of bone involvement in this population.

Further studies are needed to define both the causes and the rate of progression of bone impairment in all subtypes of SMA patients. It would be interesting to investigate the efficacy of drugs (e.g., nusinersen) and gene therapy in improving bone density and preventing fragility fractures.

Beyond classic SMN1-related SMA, “SMA plus” or atypical SMA phenotypes, characterized by a distinct pattern of weakness and specific gene mutations, other than SMN, have been recently described. SMA plus includes some variants of antenatal SMA associated with skeletal abnormalities, in which arthrogryposis, osteopenia, and multiple fractures are typically present at birth associated with severe hypotonia (23). Fetal akinesia predisposes to multiple congenital contractures and contributes to abnormal intrauterine neuromuscular development (24).

For example, a premature variant of SMA with a mutation in two genes encoding part of activating signal cointegrator 1 (ASC-1) complex, a regulator of neuromuscular unit and probably of the adjacent bony structures, is responsible for congenital bone fractures (25).

## NMDs Involving Peripheral Nerve

A typical peripheral nervous system disease that can potentially involve bone at various stages of life is the hereditary motor and sensor neuropathy or CMT.

This condition, occurring early in the first to third decade, is characterized by alterations of proteins involved in the structure and function of the peripheral nervous system; it includes axonal, demyelinating, and dominant intermediate variants, with a homogeneous clinical phenotype (26). Patients with CMT manifest symmetric, distal motor neuropathy associated with muscle weakness and atrophy, areflexia, and distal sensory loss. For CMT, there are no existing data reporting its role in bone health during fetal development. Indeed, CMT typically begins in childhood, and lower limbs are more affected, making walking increasingly difficult. Beside the sensory-motor component, in CMT patients, several bone deformities (i.e., hip dysplasia and/or cavovarus foot) occur as well as a balance impairment resulting in increased risk of falls and fractures (27). There is little evidence of a role of CMT mutated genes during embryonic and postnatal periods. Therefore, children affected by CMT seem not predisposed to secondary osteoporosis, despite the muscle weakness, and to fragility fractures although bone deformities and balance impairment occur. On the other hand, in adult CMT patients, an increased fracture risk of 1.5-fold has been reported. Also, in this population, the increased risk of fractures mainly affects distal limbs and not the sites of major osteoporotic fractures (spine, hip, proximal humerus, and wrist). Nevertheless, fractures in adults with CMT are not considered depending on low BMD and are interpreted as high-energy trauma injury, occurring in the first year after diagnosis in uncommon osteoporotic sites (i.e., ankle, hand, foot) (28). Therefore, it is likely that even for adults with CMT, primary and secondary bone damage is not a determining factor for fracture risk, while muscle weakness and sensorial alterations are likely to affect gait performance and consequent increased risk of falls. Finally, no data are reported on the alteration of bone turnover and BMD during the time span of the disease.

## NMDs Involving Neuromuscular Junction

Myasthenia gravis is an autoimmune NMD caused by autoantibodies against the acetylcholine receptor (AChR), and less frequently to MuSK protein (muscle-specific kinase) or the LRP4 (lipoprotein related protein 4), located on post-synaptic membrane of the skeletal muscles (29). Myasthenia gravis is characterized by an unexpected fluctuating clinical scenario with variable weakness distribution involving the ocular, bulbar, limb, and respiratory muscles (30). Considering the age of onset of clinical manifestation, MG is classified into juvenile MG (pre and post pubertal) and adult MG (early and late onset).

A significantly increased risk of osteoporosis in patients affected by MG has been demonstrated, with an HR of 1.52 in corticosteroid-naïve patients, whereas the corticosteroid-treated group had an HR of 2.37 (31).

Danikowski et al. hypothesized that enhanced bone resorption and myogenic proliferation inhibition in MG patients might be related to dysfunction of T-regulatory cells (Tregs) and overexpression of cytokines and chemokines, such as tumor necrosis factor-α (TNF-α), interleukin-6 (IL-6), and 10 (IL-10) (32, 33). Also genetic variants could be involved in the pathophysiological mechanisms of MG, resulting in heterogeneous phenotypes. A recent genome-wide association study of MG demonstrates the relationship between TNFRSF11A gene and MG. This gene encodes the receptor activator of nuclear factor-κ B (RANK) involved in both immune surveillance and osteoclastogenesis. In particular, mutations in TNFRSF11A are responsible for inherited forms of osteopetrosis and Paget's disease of bone and seem to be related to late-onset MG patients (34).

These data allow us to speculate on the primary bone damage that has been observed in the late-onset variant of MG associated with TNFRSF11A mutation. However, bone damage is associated with GC use in MG patients as well. Furthermore, in this population, a high incidence of vitamin D deficiency and increased serum levels of bone resorption markers (i.e., CTx) were reported (35).

While bone involvement in MG is well described in adults, particularly in GC users, two variants of MG characterized by bone damage occurring in embryonic and postnatal periods, such as congenital and neonatal myasthenia (NM), have been identified: congenital myasthenia is an autosomal recessive inherited disorder of the childhood, resulting from choline acetyltransferase (ChAT) deficiency and postsynaptic anomalies.

Neonatal myasthenia affects newborn of women with MG due to the passive transfer of maternal autoantibodies, which recognize fetal acetylcholine receptors. Neonatal myasthenia is transitory and symptoms disappear after a maximum of a few weeks when the antibody titer decreases (36).

Sometimes, newborns affected by NM could manifest arthrogryposis multiplex congenita (AMC) caused by lack of fetal movement *in utero* with consequent multiple joint contractures and growth retardation. Ultrasonography evaluation of long bones in fetuses with AMC shows a hypomineralization and hypoechogenicity of long bones with osteopenia to support the role of muscle hypotonia on bone development (37).

## NMDs Involving Skeletal Muscle Structure

A representative NMD involving skeletal muscle, characterized by genetic etiology and early disease onset, is DMD.

Duchenne muscular dystrophy is an X-linked disease caused by a mutation in the DMD gene that encodes dystrophin protein. Becker muscular dystrophy is an allelic X-linked variant of muscular dystrophy that leads to a reduction in dystrophin expression and a milder clinical phenotype (38).

In DMD, muscular impairment is symmetric and proximal, involving pelvic girdle that causes the classic clinical sign, Gower's maneuver, that indicates proximal lower limbs weakness (39).

Also, for DMD, the reduced biomechanical muscle strength and loss of independent ambulation cause bone alterations and secondary osteoporosis. In particular, structural changes at the myotendinous junctions, due to dystrophin complex alteration, are responsible for reduced force transmission and mechanical stimuli on bone tissue during muscular contraction with a detrimental effect on bone health (40). The main clinical consequences are bone fragility and higher risk of fracture. Because muscle weakness usually occurs at about 3–6 years old, in DMD patients, it is very likely that no bone damage is present at birth. Thus, we can believe that this disease interferes with muscle–bone cross-talk preferentially in the phase of allometric growth (41).

From a functional point of view, DMD children might not achieve or maintain the motor development milestones, and are compelled to use wheelchair in a few years. During the progression of disease, they became wheelchair-dependent usually at the age of 13 years with worsening of bone deformities and onset of scoliosis (42).

In association with mobility limitation, secondary osteoporosis in DMD patients is due to the prolonged use of GCs. This therapy is usually initiated at approximately 5 years of age, which has resulted in a significant improvement in the clinical and functional status, delaying the loss of independent ambulation. However, the use of deflazacort or prednisone at the dose of maximum 36 and 30 mg/day, respectively (0.75 mg/kg/day; 0.9 mg/kg/day), leads to a further worsening of bone health in terms of BMD and bone quality, through its direct and indirect effects (38). As a direct effect, GCs suppress bone formation by reducing osteoblast proliferation, differentiation, and function. GCs also influence the activity of osteocytes, mechanosensors of the bone, inducing apoptosis. Moreover, GCs increase the osteoclast activity and enhance the production of pro-inflammatory cytokines, such as IL-6. As an indirect effect, GCs inhibit gastrointestinal calcium absorption with a resultant fall in the ionized calcium concentration and the subsequent rise in serum PTH concentration. Finally, GCs play a role in the GH/IGF I axis and sex hormone axis, reducing IGF I and estrogen expression, respectively (43).

Fragility fracture risk in patients with DMD is significantly higher (44), with a fracture prevalence 5-fold higher compared with age-matched healthy young people (48 vs. 9%) with lower limb fractures (femur and tibia) more frequent in DMD. Most of the long-bone fractures in DMD patients are caused by low-energy trauma (i.e., fall from a standing position), an extremely rare condition in healthy young people. Long-bone fractures substantially contribute to functional and postural impairments, accelerating the transition to a wheelchair (45). Moreover, increased fracture risk of 75% (all skeletal sites) after 3 months of full wheelchair use has also been demonstrated (46). Finally, in GC-treated DMD patients, a moderate prevalence of symptomatic VFs (up to 40%) has been reported (47).

Joseph et al. showed a mean latency of 2.3 years to incur in symptomatic VFs after the beginning of the treatment (48). However, painful VFs are present in less than half of DMD patients, with these lesions frequently detected as asymptomatic incidental findings. Therefore, adequate follow-ups by performing spine radiograph are mandatory to have an early identification of the first fracture, as well as to monitor and prevent the occurrence of multiple VFs (“cascade fracturative”). Healthy children with VFs might show a spontaneous growing-mediated phenomenon, called vertebral reshaping, a vertebral restoration in height and width due to physiological bone remodeling. In children affected by several chronic illnesses such as DMD, the child's self-correcting reshaping mechanism fails to occur, but the administration of intravenous bisphosphonates (BPs) has been demonstrated to restore this process; therefore, when the fracture appears, antiresorptive drug treatment should start as early as possible (9, 49).

Further side effects of GC treatment are the impaired growth and delayed puberty due to hypogonadism caused by reduced testosterone serum level that compromises bone health (50). The role of testosterone on bone health is well established; direct and indirect mechanisms are mainly involved (51). First, low level of androgens causes muscle weakness that reduces mechanical stimulation on bones (52). Secondly, reduced activation of bone androgen receptor by testosterone has a negative impact on the expression and function of the all three lines of bone-related cells (osteoblast, osteoclast, and osteocyte), resulting in an overall bone loss. Testosterone is also partially converted via aromatase into estrogens that play a crucial role in peak bone mass acquisition and in periosteal bone expansion during puberty in men (53).

## NMDs Involving Skeletal Muscle Metabolism

In the context of metabolic myopathies, Pompe's disease (PD) is an inherited NMD associated with low BMD and increased risk of fracture.

Pompe's disease is an autosomal recessive lysosomal storage disorder (LSD) due to mutations that cause reduction or absence of acid α-glucosidase (GAA gene on chromosome 17). This enzyme is ubiquitous in the body, and its reduction in activity leads to a progressive accumulation of glycogen within the lysosomes, and later between myofibrils, contributing to the clinical phenotype of skeletal, cardiac, and respiratory muscle involvement (54, 55). Two main forms of PD are described: infantile onset (IOPD, classic and non-classic variants) and late onset (LOPD), different in terms of organ involvement and the rate of progression. Severe muscle weakness and cardiac involvement characterize IOPD; on the contrary, a slow progression of limb–girdle muscle weakness and a respiratory dysfunction are typical of LOPD (56).

In both the PD variants, bone health is largely compromised, predisposing to an increased risk of osteoporosis or low bone mass for chronological age, strongly correlated to reduction of muscle strength (57).

In IOPD, severe muscle wasting negatively influences bone health in early phases of skeletal development, particularly at cortical sites, and consequently is associated with a higher risk of long-bone fractures (58). On the other hand, evidence of a higher prevalence of fragility fractures in LOPD patients is limited.

Exact pathogenetic mechanisms underneath muscle and bone relationship in LOPD patients are still unknown. These patients have different patterns of muscle involvement, commonly characterized by weakness of paravertebral, hip flexor, and knee extensor muscles that results in poor mechanical load applied to the spine and the proximal femur (59). The clinical phenotype of muscle impairment might explain the site-specific BMD loss (i.e., at LS and/or femoral neck). van den Berg showed a decreased BMD particularly in non-ambulatory and ventilator-dependent patients (54 vs. 15% in ambulant subjects) (58). The authors found a statistically significant positive mild-to-moderate correlation between total body less head (TBLH) BMD and proximal muscle strength of both the upper (*r* = 0.37) and lower extremities (*r* = 0.43). Moreover, this study suggested a putative role of prolonged inactivity and immobilization as risk factors for bone loss in these patients (58). These data were confirmed by Khan et al. who analyzed bone microarchitecture using high-resolution peripheral computed tomography (HR-pQCT) at two standard skeletal sites, distal radius and distal tibia (60).

Bertoldo et al. showed that asymptomatic and atraumatic VFs occur frequently in LOPD patients. However, the authors did not find any relationship between muscle function [assessed by Medical Research Council Scale (MRC), 6-min walking test (6MWT), and Gait, Stairs, Gower and Chair score (GSGC)] and BMD or biochemical markers of bone metabolism. No difference in muscle function, respiratory parameters, and serum 25(OH)D between patients with or without morphometric VF was also reported. Therefore, the authors conclude that bone involvement seems to be independent from muscular phenotype and vitamin D status in patients with LOPD (61).

With the introduction of the enzyme replacement therapy (ERT), the course of disease has been strongly modified, in particular for the IOPD variant, improving both muscle and respiratory functions. Otherwise, tangible effects of ERT in terms of improving bone health are poorly supported (62, 63).

To date, bone damage in PD is considered a consequence of muscle atrophy and reduction of weight bearing and load; furthermore, taking into account the severity of bone involvement in IOPD, a potential intrinsic bone mechanism directly linked to metabolic degeneration itself or to lysosome storage alteration is suggested.

## Bone Health Assessment in NMDs

In the multidisciplinary and comprehensive management of patients affected by NMDs, the assessment of bone health status through clinical, instrumental, and laboratory testing should be integrated as a mandatory component in the evaluation of muscle impairment and functional independence.

Bone health screening in NMDs should include:

- Laboratory exams of bone metabolism such as serum and urinary calcium and phosphorus, serum parathyroid hormone (PTH), 25(OH)D, and alkaline phosphatase (ALP) (at the diagnosis and every 12 months);

- Imaging: lateral thoracolumbar spine radiographs at least every 12–36 months, taking into account drug therapy with detrimental effects on bone health (i.e., GCs), which require closer follow-ups. However, in case of referred low back pain, history of recent trauma, or spine BMD *Z*-score decline >0.5 SD in serial follow-ups, lateral thoracolumbar spine x-ray should be taken, to assess VFs.

- Dual-Energy X-ray Absorptiometry (DXA) to measure BMD and bone mineral content (BMC) considered as *Z*-score for children and *T*-score for adults over 50 years old and postmenopausal women (at the diagnosis and at least every 12 months). According to the International Society for Clinical Densitometry (ISCD), skeletal health assessment in children (between 5 and 19 years old) consists of DXA evaluation including posterior–anterior (PA) spine and total body less head (TBLH) scans for BMC and BMD measurements. However, these skeletal sites may not be suitable for densitometric assessment for all children affected by clinical conditions adversely involving bone health. Therefore, in recent ISCD Official Positions, additional DXA scans (proximal femur, PF, LDF, and distal forearm, DF) as well as the role of vertebral fracture assessment (VFA) were thoroughly evaluated for utility, applicability, and ability to assess skeletal health and to predict fragility fractures in selected pediatric patients (44). Indeed, for many children with NMDs with poor ambulatory status and at risk of bone damage, these additional DXA scans are useful when TBLH or LS scans are not feasible (i.e., positioning issues, severe scoliosis).

Moreover, the occurrence and identification of spine fractures remain critical issues for skeletal health in individuals at risk of poor bone strength, including NMD patients. However, the most recent generation of DXA scanners provides a better resolution of lateral spine imaging compared to the past, thus allowing a safer bone health monitoring, by minimizing radiation exposure of children affected by NMDs.

The presence of at least one vertebral fragility fracture is sufficient for definition of osteoporosis in children. Moreover, an alternative diagnostic criterion in the same population includes densitometric assessment, namely, BMD *Z*-score ≤ −2.0 SD, combined with a clinically significant history of fracture (two or more long-bone fractures by 10 years of age and/or three or more long-bone fractures up to 19 years); BMD *Z*-score ≤ −2.0 SD without a history of fractures should be defined as low BMD for age and gender (64, 65). In men younger than 50 years or premenopausal women, the ISCD recommends the use of *Z*-scores; diagnosis of low BMD in this population is defined by a *Z*-score of ≤ −2.0 SD (the term “osteoporosis” should be avoided in these cases) (66, 67). In postmenopausal women and in men aged 50 years and older, osteoporosis may be appropriately diagnosed when LS, total hip, or femoral neck *T*-score is ≤ -2.5 SD (BMD that lies −2.5 SD or more below the average value for young healthy individuals) (68).

Diagnosis of osteopenia includes LS, total hip, or femoral neck BMD *T*-score between −1.0 and −2.5 SD (Table 3) (69–73).

**Table 3:** Bone health assessment in NMDs.

	**NMDs**	**Bone turnover markers**	**DXA**	**Lateral toracolumbar spine radiograph**
SMA (types 1–4)	Sitters	At the diagnosis and every 12 months	At the diagnosis and every 12 months	^*^If symptomatic
	Non-sitters			
CMT		At the diagnosis and every 12 months	At the diagnosis and eventually every 24 months	Not required
MG	On GC	At the diagnosis and every 12 months	At the diagnosis and every 12 months	^*^If symptomatic
	Not on GC		At the diagnosis and eventually every 24 months	
DMD	On GC	At the diagnosis and every 12 months	At the diagnosis and every 12 months	Every 1–2 years
	Not on GC			Every 2–3 years
PD	IOPD	At the diagnosis and every 12 months	At the diagnosis and every 12 months	^*^If symptomatic
	LOPD			

* *Presence of back pain or suspected vertebral fractures*.

For the assessment of bone health in NMDs, nutritional assessment is advised, in particular for the pediatric population (74).

Nutrient requirement is different during the course of life, and monitoring their intake is useful also to better prevent underfeeding or overfeeding. Nutritional calcium intake, through specific questionnaires available online (https://www.iofbonehealth.org/calcium-calculator), is mandatory for bone health assessment, especially in NMDs.

Finally, sunlight exposure of a patient affected by NMD should be investigated, considering that 80% of vitamin D is produced by the skin under sunlight irradiation.

## Therapeutic Opportunities

### Non-pharmacological Therapies

Therapeutic management of patients affected by NMDs should take into account many clinical features that occur during the disease. Novel therapies available and better clinical approach to these patients lead to longer survival, allowing the observation of additional clinical issues previously poorly reported, in particular the progressive decline of bone health. Therefore, a tailored approach has to be implemented in terms of prevention and treatment of bone complications. Of prime importance, patients with NMDs should adopt a healthy lifestyle from as early an age as possible with the objective to optimally preserve musculoskeletal health. In this context, healthy lifestyle includes nutrition, sun exposure, and physical activity.

Nutrition has a pivotal role in musculoskeletal metabolism. In several NMD conditions, overnutrition is a major issue caused by an excess of caloric intake. The use of some drugs, such as corticosteroids (e.g., DMD patients), leads to an increased appetite, and the compassionate attitude of the caregiver could significantly contribute to weight gain (74). On the other hand, undernutrition is more common in severe and advanced stages of different NMDs due to dysphagia, delayed gastric emptying, prolonged mealtime, and constipation, all of them related to progressive muscle weakness (75). Therefore, an adequate caloric intake is mandatory in each stage of NMDs taking into account the proper proportion between protein and complex carbohydrates (76). A calcium-rich diet based on dairy products is strongly suggested, with a calcium intake of at least 1300 mg/day in patients aged 9–18 years, and 1000 mg day between 19 and 50 years old. The recommended dose of vitamin D for infants and children aged 0–1 year is at least 400 IU/day, while in children aged 1–18 years and in adults, the recommended dose is 600 IU/day (77). When patients do not reach the suggested intakes, it is necessary to consider calcium and vitamin D supplementations. Vitamin D status that requires supplementation in children is still debated; the Institute of Medicine (IOM) suggests serum 25(OH)D <20 ng/ml as threshold to start treatment, which is also the same recommendation to initiate treatment for adults (78).

Vitamin D supplementation is usually provided by cholecalciferol administration. However, in obese patients, particularly those treated with corticosteroids, higher doses of vitamin D are needed because of low bioavailability (79). Moreover, NMD patients usually receive other drugs metabolized in the liver by the CYP system involved in the hydroxylation of native vitamin D (e.g., corticosteroids and β-blockers); therefore, the use of calcifediol, a hydroxylated metabolite of vitamin D3 with higher bioavailability that does not require hepatic activation, should be considered. Furthermore, calcifediol is usually recommended in patients with malabsorption or hepatic disease, and when a rapid increase in serum 25(OH)D is required.

In patients affected by DMD, calcifediol administration at the dose of 0.8 mcg/kg daily per os plus adjustment of the dietary calcium intake, at least 1,000 mg/day, demonstrates a statistically significant increase of BMC and BMD in over 65% of patients and normalization of bone turnover markers (BTMs) in most patients (78.8%) (80). Therefore, periodic screening of the serum 25(OH)D is strongly suggested to monitor and, if indicated, adjust the supplementation of vitamin D. At the same time, it is possible to suggest a main role of vitamin D supplementation in the management of bone metabolism disorders in other NMDs. Moreover, considering the detrimental effects of vitamin D deficiency on muscle performance (81) as well as the encouraging data supporting calcifediol use for improving muscle function in different populations (82), a putative beneficial role of vitamin D administration on skeletal muscle in NMDs has been hypothesized. Indeed, Karam et al. found an improvement of functional parameter in patients with ALS treated with high dose of cholecalciferol (83), although these results are not confirmed by a successive study (84).

Physical activity (PA) should be considered a cornerstone for the prevention and treatment of bone alterations in patients affected by NMDs. Despite the lack of evidence about the role of exercise on improving bone health in NMDs, the benefits of a tailored exercise program can be assumed.

Physical activity has a role in stimulating osteoblast bone formation and reducing osteoclast-mediated bone resorption through biological pathways modulated by mechanical loading (85). In particular, PA enhances attaining peak bone mass in childhood and adolescence, and contributes to bone turnover in adulthood (86). According to the WHO recommendation, children should perform PA for at least 60 min each day (87); however, most of NMD patients, even in the early ambulatory stages, do not reach these levels of PA (88).

Starting adequate PA as early as possible, considered as more than 3,000 accelerometer movement counts per minute (89) (about 5 metabolic equivalents, METs, corresponding to normal walking speed in children) (90), results in an improvement of 7 and 5% for the spine, and 6 and 4% for hip at 3 and 6 years, respectively, in terms of BMC (91). In patients with NMDs, dynamic low-moderate intensity weight-bearing exercises (i.e., walking) that result in greater gains in bone tissue compared to static activities are strongly recommended (92). There is no evidence that performing high-intensity exercise provides greater benefit than performing moderate-intensity exercise; on the contrary, NMD patients should avoid heavy exercises to prevent fatigue (93). In DMD patients, strengthening exercises, such arms and legs cycle in 30-min sessions (15 min for arms and 15 min for legs), 5 days per week, reduce muscle disuse atrophy and excessive functional loss (94).

Moreover, balance disorders are frequently reported in patients with NMDs. In particular, it has been demonstrated that patients with proximal muscle weakness (i.e., DMD) show more difficulties in static balance, while patients with primarily distal muscle involvement (i.e., CMT) show more difficulties in dynamic balance. Exercises to maintain muscle strength that improve balance when performing both static and dynamic activities reduce the risk of fall (95).

### Pharmacological Therapies

#### Bisphosphonates

In association with a proper lifestyle, with the aim to improve bone strength and reduce the risk of fractures, the use of pharmacological treatment is advisable. Anti-osteoporotic drugs, such as BPs, are widely used in postmenopausal and GC-induced osteoporosis (GIO). Bisphosphonates have a high affinity to bone tissue, and they are capable of inhibiting bone resorption in humans, mainly leading to osteoclast apoptosis with different mechanisms of action (96). The use of antiresorptive drugs has been already investigated in children; in particular, BPs have been demonstrated to have efficacy and safety in patients affected by other rare diseases, such as Paget disease of bone (97, 98), osteogenesis imperfecta (OI) (99), β-thalassemia (100), and overall secondary osteoporosis in children (101). Limited evidence supports the use of BPs in NMDs. To date, an abstract recently presented at the 101st Annual Meeting of the Endocrine Society including four children affected by SMA on intravenous BPs therapy (102) showed no significant change in BMD at 1 year follow-up; the authors also reported some mild adverse events and one atypical femur fracture. In MG, the use of oral alendronate (70 mg/week) in a patient under GC treatment improved the LS BMD of 3.4% after 12 months without side effects (103). However, caution should be paid for possible exacerbation of MG symptoms, such as muscle weakness and extreme fatigue, after drug administration (104).

In a case report of an osteoporotic patient, symptoms of MG were exacerbated by the administration of risedronate (105); however, the exact mechanism underneath this pathological condition remains unclear.

Recent care recommendations endorse the use of intravenous BPs as first-line therapy for the treatment of osteoporosis in DMD patients with fragility fractures of the spine or long bones (72, 106). Moreover, the administration of oral alendronate or risedronate (107) and intravenous pamidronate or zoledronic acid (106) seems to reduce back pain in patients affected by VF. The role of BPs for primary prevention of fragility fractures in DMD as for the other NMDs is still debated.

### Denosumab

Denosumab (Dmab), a fully human IgG2 monoclonal antibody that neutralizes the receptor activator of nuclear factor kappa-B ligand (RANKL), is the most potent antiresorptive agent available in clinical practice. It blocks the interaction between the cytokine and its receptor (RANK), inhibiting osteoclast-mediated bone resorption. Approved by the FDA for postmenopausal osteoporotic women at high risk of fracture, Dmab reduces the risk of vertebral and non-vertebral fractures compared to placebo (108) and improves BMD compared to BPs (109).

To the best of our knowledge, few anecdotal studies demonstrate the effectiveness of Dmab in NMDs.

A case report in a 14-year-old patient affected by SMA II showed an improvement of LS BMD of 19% after 6 months of 60 mg s.c. of Dmab without adverse effects (110). The same dosage of Dmab increased significantly LS BMD (about 16%) at 12 months in a 13-year-old boy with DMD and GIO (111).

### Teriparatide

Teriparatide is a recombinant human parathyroid hormone 1–34 (rhPTH 1–34) stimulating bone formation. It is widely used in postmenopausal women at high risk of fracture and in GIO (112). Despite its potential benefits, in particular on trabecular bone in NMD patients with VF on GC therapy, its use is reserved for selected patients. In particular, the safety of teriparatide has not been established in pediatric patients due to the high risk of osteosarcoma (113). However, to date, a single case report shows improvement of the LS *Z*-score BMD, back pain, and quality of life in a DMD patient with multiple VFs in GC treatment after the administration of 20 mcg once daily of s.c. teriparatide (114).

## Conclusions

NMDs are a group of disorders characterized by skeletal muscle wasting that usually affect different organs and systems, including bone. Muscle–bone cross-talk plays a key role in the pathophysiological mechanisms that result in predictable clinical phenotypes. However, this relationship is often underestimated until a fracture occurs. Instead, it is essential that the biological and functional link between muscle and bone is investigated across the three phases of bone development, including the embryonic life, the postnatal period, and the adult life. Physicians who treat adult patients with NMDs with chronic GCs should consider the administration of anti-osteoporotic medications to prevent fragility fractures, although no such formal recommendation currently exists for pediatric patients.

## Author Contributions

GI and AM contributed to conception and design of the review. MP, SL, and CC wrote the first draft of the manuscript. All authors wrote sections of the manuscript, contributed to manuscript revision, and read and approved the submitted version.

### Conflict of Interest

The authors declare that the research was conducted in the absence of any commercial or financial relationships that could be construed as a potential conflict of interest.

## References

[1] Qin Y Peng Y Zhao W Pan J Ksiezak-Reding H Cardozo C. Myostatin inhibits osteoblastic differentiation by suppressing osteocyte-derived exosomal microRNA-218: a novel mechanism in muscle-bone communication. J Biol Chem. (2017) 292:11021–11033. 10.1074/jbc.M116.770941 28465350 PMC5491785

[2] Colaianni G Mongelli T Cuscito C Pignataro P Lippo L Spiro G. Irisin prevents and restores bone loss and muscle atrophy in hind-limb suspended mice. Sci Rep. (2017) 7:2811. 10.1038/s41598-017-02557-8 28588307 PMC5460172

[3] Colaianni G Cuscito C Mongelli T Pignataro P Buccoliero C Liu P. The myokine irisin increases cortical bone mass. Proc Natl Acad Sci USA. (2015) 112:12157–62. 10.1073/pnas.1516622112 26374841 PMC4593131

[4] Kitase Y Vallejo JA Gutheil W Vemula H Jähn K Yi J. β-aminoisobutyric Acid, l-BAIBA, is a muscle-derived osteocyte survival factor. Cell Rep. (2018) 22:1531–44. 10.1016/j.celrep.2018.01.041 29425508 PMC5832359

[5] Subbotina E Sierra A Zhu Z Gao Z Koganti SR Reyes S. Musclin is an activity-stimulated myokine that enhances physical endurance. Proc Natl Acad Sci USA. (2015) 112:16042–7. 10.1073/pnas.1514250112 26668395 PMC4702977

[6] Kimonis V Inclusion body myopathy with paget disease of bone and/or frontotemporal Dementia. In: Adam MP Ardinger HH Pagon RA Wallace SE Bean LJH Stephens K Amemiya A, editors. GeneReviews®. Seattle, WA: University of Ashington (2007). p. 1993–2019.

[7] Karasik D Kiel DP. Genetics of the musculoskeletal system: a pleiotropic approach. J Bone Miner Res. (2008) 23:788–802. 10.1359/jbmr.080218 18269309 PMC3280426

[8] Feldman EL Callaghan BC Pop-Busui R Zochodne DW Wright DE Bennett DL Diabetic neuropathy. Nat Rev Dis Primers. (2019) 5:41 10.1038/s41572-019-0092-1 31197153

[9] Emery AE. Population frequencies of inherited neuromuscular diseases–a world survey. Neuromuscul Disord. (1991) 1:19–29. 10.1016/0960-8966(91)90039-U 1822774

[10] Deenen JC Horlings CG Verschuuren JJ Verbeek AL van Engelen BG. The epidemiology of neuromuscular disorders: a comprehensive overview of the literature. J Neuromuscul Dis. (2015) 2:73–85. 10.3233/JND-140045 28198707

[11] Larkindale J Yang W Hogan PF Simon CJ Zhang Y Jain A. Cost of illness for neuromuscular diseases in the United States. Muscle Nerve. (2014) 49:431–8 10.1002/mus.23942 23836444

[12] Available, online at: https://www.cdc.gov/ncbddd/disabilityandhealth/data-highlights.html

[13] Ryder S Leadley RM Armstrong N Westwood M de Kock S Butt T. The burden, epidemiology, costs and treatment for Duchenne muscular dystrophy: an evidence review. Orphanet J Rare Dis. (2017) 12:79. 10.1186/s13023-017-0631-3 28446219 PMC5405509

[14] Wasserman HM Hornung LN Stenger PJ Rutter MM Wong BL Rybalsky I. Low bone mineral density and fractures are highly prevalent in pediatric patients with spinal muscular atrophy regardless of disease severity. Neuromuscul Disord. (2017) 27:331–7. 10.1016/j.nmd.2017.01.019 28258940 PMC6448139

[15] Ma J McMillan HJ Karagüzel G Goodin C Wasson J Matzinger MA. The time to and determinants of first fractures in boys with Duchenne muscular dystrophy. Osteoporos Int. (2017) 28:597–608. 10.1007/s00198-016-3774-5 27774565

[16] Arnold WD Kassar D Kissel JT. Spinal muscular atrophy: diagnosis and management in a new therapeutic era. Muscle Nerve. (2015) 51:157–67. 10.1002/mus.24497 25346245 PMC4293319

[17] Kurihara N Menaa C Maeda H Haile DJ Reddy SV. Osteoclast-stimulating factor interacts with the spinal muscular atrophy gene product to stimulate osteoclast formation, J Biol Chem. (2001) 276:41035–9. 10.1074/jbc.M100233200 11551898

[18] Shanmugarajan S. Congenital bone fractures in spinal muscular atrophy: functional role for SMN protein in bone remodeling. J Child Neurol. (2007) 22:967–73. 10.1177/0883073807305664 17761651 PMC2787099

[19] Prior TW Finanger E Spinal muscular atrophy. In: Adam MP Ardinger HH Pagon RA Wallace SE Bean LJH Stephens K Amemiya A, editors. GeneReviews®. Seattle, WA: University of Washington (2000). p. 1993–2019. 20301526

[20] Singh A Dalal P Singh J Tripathi P. Type 0 spinal muscular atrophy in rare association with congenital contracture and generalized osteopenia. Iran J Child Neurol. (2018) 12:105–8. 29379570 PMC5760681

[21] Baranello G Vai S Broggi F Masson R Arnoldi MT Zanin R. Evolution of bone mineral density, bone metabolism and fragility fractures in Spinal Muscular Atrophy (SMA) types 2 and 3. Neuromuscul Disord. (2019) 29:525–32. 10.1016/j.nmd.2019.06.001 31266719

[22] Vai S Bianchi ML Moroni I Mastella C Broggi F Morandi L. Bone and spinal muscular atrophy. Bone. (2015) 79:116–20. 10.1016/j.bone.2015.05.039 26055105

[23] Teoh HL Carey K Sampaio H Mowat D Roscioli T Farrar M. Inherited paediatric motor neuron disorders: beyond spinal muscular atrophy. Neural Plast. (2017) 2017:6509493. 10.1155/2017/6509493 28634552 PMC5467325

[24] Bamshad M Van Heest AE Pleasure D. Arthrogryposis: a review and update. J Bone Joint Surg Am. (2009) 91(Suppl. 4):40–6. 10.2106/JBJS.I.00281 19571066 PMC2698792

[25] Knierim E Hirata H Wolf NI Morales-Gonzalez S Schottmann G Tanaka Y. Mutations in subunits of the activating signal cointegrator 1 complex are associated with prenatal spinal muscular atrophy and congenital bone fractures. Am J Hum Genet. (2016) 98: 473–89. 10.1016/j.ajhg.2016.01.006 26924529 PMC4800037

[26] Vallat JM Mathis S Funalot B. The various Charcot-Marie-Tooth diseases. Curr Opin Neurol. (2013) 26:473–80. 10.1097/WCO.0b013e328364c04b 23945280

[27] Bird TD Charcot-Marie-Tooth (CMT) hereditary neuropathy overview. In: Adam MP Ardinger HH Pagon RA Wallace SE Bean LJH Stephens K Amemiya A, editors. GeneReviews®. (1998).

[28] Pouwels S de Boer A Leufkens HG Weber WE Cooper C de Vries F Risk of fracture in patients with Charcot-Marie-Tooth disease, Muscle Nerve. (2014) 50:919–24. 10.1002/mus.24240 24634316

[29] Gilhus NE Verschuuren JJ. Myasthenia gravis: subgroup classification and therapeutic strategies. Lancet Neurol. (2015) 14:1023–36. 10.1016/S1474-4422(15)00145-3 26376969

[30] Berrih-Aknin S Frenkian-Cuvelier M Eymard B. Diagnostic and clinical classification of autoimmune myasthenia gravis. J Autoimmun. (2014) 48–49:143–8. 10.1016/j.jaut.2014.01.003 24530233

[31] Yeh JH Chen HJ Chen YK Chiu HC Kao CH. Increased risk of osteoporosis in patients with myasthenia gravis: a population-based cohort study. Neurology. (2014) 83:1075–9. 10.1212/WNL.0000000000000804 25122205

[32] Danikowski KM Jayaraman S Prabhakar BS. Regulatory T cells in multiple sclerosis and myasthenia gravis. Neuroinflammation. (2017) 14:117. 10.1186/s12974-017-0892-8 28599652 PMC5466736

[33] Meyer SU Krebs S Thirion C Blum H Krause S Pfaffl MW. Tumor necrosis factor alpha and insulin-like growth factor 1 induced modifications of the gene expression kinetics of differentiating skeletal muscle cells. PLoS ONE. (2015) 10:e0139520 10.1371/journal.pone.0139520 26447881 PMC4598026

[34] Renton AE Pliner HA Provenzano C Evoli A Ricciardi R Nalls MA. A genome-wide association study of myasthenia gravis. JAMA Neurol. (2015) 72:396–404. 10.1001/jamaneurol.2014.4103 25643325 PMC4856525

[35] Guan Y Lv F Meng Y Ma D Xu X Song Y. Association between bone mineral density, muscle strength, and vitamin D status in patients with myasthenia gravis: a cross-sectional study. Osteoporosis Int. (2017) 28:2383–90. 10.1007/s00198-017-4041-0 28439619

[36] Peragallo JH. Pediatric Myasthenia Gravis. Sem Pediatr Neurol. (2017) 24:116–21. 10.1016/j.spen.2017.04.003 28941526

[37] Murphy JC Neale D Bromley B Benacerra BR Copel JA. Hypoechogenicity of fetal long bones: a new ultrasound marker for arthrogryposis. Prenat Diagn. (2002) 22:1219–22. 10.1002/pd.492 12478637

[38] Darras BT Urion DK Ghosh PS. Dystrophinopathies, dystrophinopathies. In: Adam MP Ardinger HH Pagon RA Wallace SE Bean LJH Stephens K Amemiya A, editors. GeneReviews® (2000).20301298

[39] Guzmán ORC García ALC Cruz MR Muscular dystrophies at different ages: metabolic and endocrine alterations. Int J Endocrinol. (2012) 2012:485376 10.1155/2012/485376 22701119 PMC3371686

[40] Welser JV Rooney JE Cohen NC Gurpur PB Singer CA Evans RA. Myotendinous junction defects and reduced force transmission in mice that lack alpha7 integrin and utrophin. Am J Pathol. (2009) 175:1545–54. 10.2353/ajpath.2009.090052 19729483 PMC2751551

[41] Sarrazin E von der Hagen M Schara U von Au K Kaindl AM. Growth and psycomotor development of patients with Duchenne muscular dystrophy. Eur J Pediatr Neurol. (2014) 18:38–44. 10.1016/j.ejpn.2013.08.008 24100172

[42] Perera N Farrar M Bone health in children with duchenne muscular dystrophy: a review. Pediat Therapeut. (2015) 5:252 10.4172/2161-0665.1000252

[43] Canalis E Mazziotti G Giustina A Bilezikian JP. Glucocorticoid-induced osteoporosis: pathophysiology and therapy. Osteoporos Int. (2007) 18:1319–28. 10.1007/s00198-007-0394-0 17566815

[44] Weber DR Boyce A Gordon C Högler W Kecskemethy HH Misra M. The utility of DXA assessment at the forearm, proximal femur, and lateral distal femur, and vertebral fracture assessment in the pediatric population: the 2019 official pediatric positions of the ISCD. J Clin Densitom. (2019). 10.1016/j.jocd.2019.07.002. [Epub ahead of print]. 31421951 PMC7010480

[45] Ness K Apkon SD. Bone health in children with neuromuscular disorders. J Pediatr Rehabil Med. (2014) 7:133–42. 10.3233/PRM-140282 25096865

[46] James KA Cunniff C Apkon SD Mathews K Lu Z Holtzer C Risk factors for first fractures among males with duchenne or becker muscular dystrophy. J Pediatr Orthopaed. 35:640–44. 10.1097/BPO.0000000000000348 25379822

[47] Buckner JL Bowden SA Mahan JD. optimizing bone health in duchenne muscular dystrophy. Int J Endocrinol. (2015) 2015:928385 10.1155/2015/928385 26124831 PMC4466394

[48] Joseph S Wang C Di Marco M Horrocks I Abu-Arafeh I Baxter A. Fractures and bone health monitoring in boys with Duchenne muscular dystrophy managed within the Scottish Muscle Network. Neuromuscul Disord. (2019) 29:59–66 10.1016/j.nmd.2018.09.005 30473133

[49] Petty R Laxer RM Lindsley CB Wedderburn LR (eds.). Textbook of Pediatric Rheumatology, 7^th^ edn. Philadelphia: Elsevier (2016).

[50] Birnkrant DJ Bushby K Bann CM Apkon SD Blackwell A Brumbaugh D DMD Care Considerations Working Group, Diagnosis and management of Duchenne muscular dystrophy, part 1: diagnosis, and neuromuscular, rehabilitation, endocrine, and gastrointestinal and nutritional management. Lancet Neurol. (2018) 17:251–67. 10.1016/S1474-4422(18)30024-3 29395989 PMC5869704

[51] Golds G Houdek D Arnason T. Male hypogonadism and osteoporosis: the effects, clinical consequences, and treatment of testosterone deficiency in bone health. Int J Endocrinol. (2017) 2017:4602129. 10.1155/2017/4602129 28408926 PMC5376477

[52] Kao KT Joseph S Capaldi N Brown S Di Marco M Dunne J. Skeletal disproportion in glucocorticoid-treated boys with Duchenne muscular dystrophy. Eur J Pediatr. (2019) 178:633–40. 10.1007/s00431-019-03336-5 30762116 PMC6459782

[53] Vanderschueren D Laurent MR Claessens F Gielen E Lagerquist MK Vandenput L. Sex steroid actions in male bone. Endocr Rev. (2014) 35:906–60. 10.1210/er.2014-1024 25202834 PMC4234776

[54] Hirschhorn R Reuser A Glycogen storage disease type II: acid alphaglucosidase (acid maltase) deficiency. In: Scriver C Beaudet A Sly W Valle D, editors. The Metabolic and Molecular Bases of Inherited Disease. 8th ed New York, NY: McGraw-Hill (2001). p. 3389–420.

[55] Kishnani PS Steiner RD Bali D Berger K Byrne BJ Case LE. Pompe disease diagnosis and management guideline. Genet Med. (2006) 8:267–88. 10.1097/01.gim.0000218152.87434.f3 16702877 PMC3110959

[56] Katzin LW Amato AA. Pompe disease: a review of the current diagnosis and treatment recommendations in the Era of enzyme replacement therapy. J Clin Neuromusc Dis. (2008) 9:421–31. 10.1097/CND.0b013e318176dbe4 18525427

[57] van den Berg LE Zandbergen AA van Capelle CI de Vries JM Hop WC van den Hout JM. Low bone mass in Pompe disease: muscular strength as a predictor of bone mineral density. Bone. (2010) 47:643–9. 10.1016/j.bone.2010.06.021 20601298

[58] van den Berg LEM The Musculoskeletal System in Pompe Disease: Pathology, Consequences and Treatment Options. Erasmus University Rotterdam (2014). Available online at: http://hdl.handle.net/1765/51599

[59] Case LE Kishnani PS. Physical therapy management of Pompe disease. Genet Med. (2006) 8 318–27. 10.1097/01.gim.0000217789.14470.c5 16702883

[60] Khan A Weinstein Z Hanley DA Casey R McNeil C Ramage B. *In vivo* bone architecture in Pompe disease using high-resolution peripheral computed tomography. JIMD Rep. (2013) 7:81–8. 10.1007/8904_2012_146 23430500 PMC3575046

[61] Bertoldo F Zappini F Brigo M Moggio M Lucchini V Angelini C. Prevalence of asymptomatic vertebral fractures in late-onset pompe disease. J Clin Endocrinol Metab. (2015) 100:401–6. 10.1210/jc.2014-2763 25396301

[62] Papadimas G Terzis G Papadopoulos C Areovimata A Spengos K Kavouras S. Bone density in patients with late onset pompe disease. Int J Endocrinol Metab. (2012) 10:599–603. 10.5812/ijem.4967 23843830 PMC3693639

[63] Sheng B Chu YP Wong WT Yau EKC Chen SPL Luk WH. Improvement of bone mineral density after enzyme replacement therapy in Chinese late-onset Pompe disease patients. BMC Res Notes. (2017) 10:351. 10.1186/s13104-017-2681-y 28754168 PMC5534128

[64] Gordon CM Leonard MB Zemel BS. 2013 Pediatric Position Development Conference: executive summary and reflections. J Clin Densitom. (2014) 17:219–24. 10.1016/j.jocd.2014.01.007 24657108

[65] Yasar E Adigüzel E Arslan M Matthews DJ. Basics of bone metabolism and osteoporosis in common pediatric neuromuscular disabilities. Eur J Paediatr Neurol. (2018) 22:17–26. 10.1016/j.ejpn.2017.08.001 28830650

[66] Papaioannou A Morin S Cheung AM Atkinson S Brown JP Feldman S Scientific Advisory Council of Osteoporosis Canada; 2010 clinical practice guidelines for the diagnosis and management of osteoporosis in Canada: summary. CMAJ. (2010) 182:1864–73. 10.1503/cmaj.100771 20940232 PMC2988535

[67] Shepherd JA Schousboe JT Broy SB Engelke K Leslie WD. Executive summary of the 2015 ISCD position development conference on advanced measures From DXA and QCT: fracture prediction beyond BMD. J Clin Densitom. (2015) 18:274–86. 10.1016/j.jocd.2015.06.013 26277847

[68] Jeremiah MP Unwin BK Greenawald MH Casiano VE. Diagnosis and management of osteoporosis. Am Fam Physician. (2015) 92:261–8. 26280231

[69] World Health Organization (WHO) WHO Scientific Group on the Assessment of Osteoporosis at Primary Health Care Level: Summary Meeting Report. (2019). Available online at: http://www.who.int/chp/topics/Osteoporosis.pdf (accessed August 25, 2019).

[70] Mercuri E Finkel RS Muntoni F Wirth B Montes J Main M SMA Care Group, Diagnosis and management of spinal muscular atrophy: Part 1: recommendations for diagnosis, rehabilitation, orthopedic and nutritional care. Neuromuscul Disord. (2018) 28:103–15. 10.1016/j.nmd.2017.11.005 29290580

[71] Ilias I Zoumakis E Ghayee H An overview of glucocorticoid induced osteoporosis. In: Feingold KR Anawalt B Boyce A Chrousos G Dungan K Grossman A Hershman JM Kaltsas G Koch C Kopp P Korbonits M McLachlan R Morley JE New M Perreault L Purnell J Rebar R Singer F Trence DL Vinik A Wilson DP, editors. Endotext (2018).

[72] Birnkrant DJ Bushby K Bann CM. Diagnosis and management of Duchenne muscular dystrophy, part 2: respiratory, cardiac, bone health, and orthopaedic management. Lancet Neurol. (2018) 17:347–61. 10.1016/S1474-4422(18)30025-5 29395990 PMC5889091

[73] Cupler EJ Berger KI Leshner RT Wolfe GI Han JJ Barohn RJ the AANEM consensus committee on late-onset pompe disease, consensus treatment recommendations for late-onset pompe disease. Muscle Nerve. (2012) 45:319–33. 10.1002/mus.22329 22173792 PMC3534745

[74] Salera S Menni F Moggio M Guez S Sciacco M Esposito S. Nutritional challenges in duchenne muscular dystrophy. Nutrients. (2017) 9:594. 10.3390/nu9060594 28604599 PMC5490573

[75] Messina S Pane M De Rose P Vasta I Sorleti D Aloysius A. Feeding problems and malnutrition in spinal muscular atrophy type II. Neuromuscul. Disord. (2008) 18:389–93. 10.1016/j.nmd.2008.02.008 18420410

[76] Bianchi ML Biggar D Bushby K Rogol AD Rutter MM Tseng B Endocrine aspect of Duchenne muscular dystrophy. Neuromuscul Disord. (2011) 21:298–303. 10.1016/j.nmd.2011.02.006 21353552

[77] Holick MF Binkley NC Bischoff-Ferrari HA Gordon CM Hanley DA Heaney RP. Endocrine society. evaluation, treatment, and prevention of vitamin d deficiency: an endocrine society clinical practice guideline. Clin Endocrinol Metab. (2011) 96:1911–30. *J Clin Endocrinol Metab.* (2011) 96:3908. 10.1210/jc.2011-0385 21646368

[78] Institute of Medicine (US) Committee to Review Dietary Reference Intakes for Vitamin D and Calcium. In: Ross AC Taylor CL Yaktine AL Del Valle HB, editors. Dietary Reference Intakes for Calcium and Vitamin D. Washington, DC: National Academies Press (2011). p. 365–402. 21796828

[79] Bian Q McAdam L Grynpas M Mitchell J Harrington J. Increased rates of Vitamin D insufficiency in boys with duchenne muscular dystrophy despite higher Vitamin D3 supplementation. Glob Pediatr Health. (2019) 6:2333794X19835661. 10.1177/2333794X19835661 30906820 PMC6421611

[80] Bianchi ML Morandi L Andreucci E Vai S Frasunkiewicz J Cottafava R. Low bone density and bone metabolism alterations in Duchenne muscular dystrophy: response to calcium and vitamin D treatment. Osteoporos Int. (2011) 22:529–39. 10.1007/s00198-010-1275-5 20458570

[81] Iolascon G Mauro GL Fiore P Cisari C Benedetti MG Panella L. Can vitamin D deficiency influence muscle performance in postmenopausal women? A multicenter retrospective study. Eur J Phys Rehabil Med. (2018) 54:676–82. 10.23736/S1973-9087.17.04533-6 28696084

[82] Iolascon G Moretti A de Sire A Calafiore D Gimigliano F. Effectiveness of calcifediol in improving muscle function in post-menopausal women: a prospective cohort study. Adv Ther. (2017) 34:744–52. 10.1007/s12325-017-0492-0 28205055

[83] Karam C Barrett MJ Imperato T MacGowan DJ Scelsa S. Vitamin D deficiency and its supplementation in patients with amyotrophic lateral sclerosis. J Clin Neurosci. (2013) 20:1550–3. 10.1016/j.jocn.2013.01.011 23815870

[84] Libonati L Onesti E Gori M.C Ceccanti M Cambieri C Fabbri A. VitaminD in amyotrophic lateral sclerosis. Funct. Neurol. (2017) 32:35–40. 10.11138/FNeur/2017.32.1.035 28380322 PMC5505528

[85] Takata S Yasui N. Disuse osteoporosis. J Med Investig. (2001) 48:147–56. 10.1016/s0140-6736(63)91031-6 11694954

[86] Weaver CM Gordon CM Janz KF Kalkwarf HJ Lappe JM Lewis R The National Osteoporosis Foundation's position statement on peak bone mass development and lifestyle factors: a systematic review and implementation recommendations. Osteoporos Int. (2016) 27:1281–386. 10.1007/s00198-015-3440-3 26856587 PMC4791473

[87] World Health Organization Global Recommendations on Physical Activity for Health. Geneva: WHO (2010). 26180873

[88] Heutinck L Kampen NV Jansen M Groot IJ. Physical activity in boys with duchenne muscular dystrophy is lower and less demanding compared to healthy boys. J Child Neurol. (2017) 32:450–7. 10.1177/0883073816685506 28112012

[89] Pulsford RM Cortina-Borja M Rich C Kinnafick FE Dezateux C Griffiths LJ. Actigraph accelerometer-defined boundaries for sedentary behaviour and physical activity intensities in 7 year old children. PLoS ONE. (2011) 6:e21822 10.1371/journal.pone.0021822 21853021 PMC3154898

[90] Evenson KR Catellier DJ Gill K Ondrak KS McMurray RG. Calibration of two objective measures of physical activity for children. J Sports Sci. (2008) 26:1557–65. 10.1080/02640410802334196 18949660

[91] Janz KF Letuchy EM Eichenberger Gilmore JM Burns TL Torner JC Willing MC. Early physical activity provides sustained bone health benefits later in childhood. Med Sci Sports Exerc. (2010) 42:1072–8. 10.1249/MSS.0b013e3181c619b2 19997029 PMC2874089

[92] Bell JM Shields MD Watters J Hamilton A Beringer T Elliott M. Interventions to prevent and treat corticosteroid-induced osteoporosis and prevent osteoporotic fractures in Duchenne muscular dystrophy. Cochrane Database Syst Rev. (2017) 1:CD010899. 10.1002/14651858.CD010899.pub2 28117876 PMC6464928

[93] Abresch RT Carter GT Han J McDonald CM. Exercise in neuromuscular diseases. Phys Med Rehabil Clin N Am. (2012) 23:653–73. 10.1016/j.pmr.2012.06.001 22938880

[94] Jansen M de Groot IJ van Alfen N Geurts ACh. Physical training in boys with Duchenne muscular dystrophy: the protocol of the No Use is Disuse study. BMC Pediatr. (2010) 10:55. 10.1186/1471-2431-10-55 20691042 PMC2929216

[95] Kaya P Alemdaroglu I Yilmaz Ö Karaduman A Topaloglu H. Effect of muscle weakness distribution on balance in neuromuscular disease. Pediatr Int. (2015) 57:92–7. 10.1111/ped.12428 24978611

[96] Russell RG Watts NB Ebetino FH Rogers MJ Mechanisms of action of bisphosphonates: similarities and differences and their potential influence on clinical efficacy. Osteoporos Int. (2008) 19:733–59. 10.1007/s00198-007-0540-8 18214569

[97] Merlotti D Rendina D Gennari L Mossetti G Gianfrancesco F Martini G. Comparison of intravenous and intramuscular neridronate regimens for the treatment of Paget disease of bone. J Bone Miner Res. (2011) 26:512–8. 10.1002/jbmr.237 20814970

[98] Merlotti D Gennari L Martini G Valleggi F De Paola V Avanzati A. Comparison of different intravenous bisphosphonate regimens for Paget's disease of bone. J Bone Miner Res. (2007) 22:1510–7. 10.1359/jbmr.070704 17605632

[99] Dwan K Phillipi CA Steiner RD Basel D. Bisphosphonate therapy for osteogenesis imperfecta. Cochrane Database Syst Rev. (2016) 10:CD005088. 10.1002/14651858.CD005088.pub4 27760454 PMC6611487

[100] Bhardwaj A Swe KM Sinha NK Osunkwo I. Treatment for osteoporosis in people with ß-thalassaemia. Cochrane Database Syst Rev. (2016) 3:CD010429. 10.1002/14651858.CD010429.pub2 26964506

[101] Ward L Tricco AC Phuong P Cranney A Barrowman N Gaboury I Bisphosphonate therapy for children and adolescents with secondary osteoporosis. Cochrane Database Syst Rev. (2007) 4:CD005324 10.1002/14651858.CD005324.pub2 PMC8884163 17943849

[102] Nasomyont N Hornung L Wasserman H Intravenous bisphosphonate therapy in children with spinal muscular atrophy. J Endocr Soc. (2019) 3(Suppl 1): SAT-LB052. 10.1210/js.2019-SAT-LB052 31788718

[103] Lv F Guan Y Ma D Xu X Song Y Li L. Effects of alendronate and alfacalcidol on bone in patients with MG initiating glucocorticoids treatment. Clin Endocrinol. (2018) 88:380–7. 10.1111/cen.13537 29266368

[104] Kesikburun S Güzelküçük U Alay S Yavuz F Tan AK. Exacerbation of myasthenia gravis by alendronate. Osteoporos Int. (2014) 25:2319–20. 10.1007/s00198-014-2768-4 24935165

[105] Raja V Sandanshiv P Neugebauer M. Risedronate induced transient ocular myasthenia. J Postgrad Med. (2007) 53:274–5. 10.4103/0022-3859.37525 18097124

[106] Sbrocchi AM Rauch F Jacob P McCormick A McMillan HJ Matzinger MA. The use of intravenous bisphosphonate therapy to treat vertebral fractures due to osteoporosis among boys with Duchenne muscular dystrophy. Osteoporos Int. (2012) 23:2703–11. 10.1007/s00198-012-1911-3 22297733

[107] Wood CL Bettolo CM Bushby K Straub V Rawlings D Sarkozy A Bisphosphonate use in Duchenne Muscular Dystrophy – why, when to start and when to stop? Expert Opinion on Orphan Drugs. (2016) 4:407–16. 10.1517/21678707.2016.1148596

[108] Cummings SR San Martin J McClung MR Siris ES Eastell R Reid IR. Denosumab for prevention of fractures in postmenopausal women with osteoporosis. N Engl J Med. (2009) 361:756–65. 10.1056/NEJMoa0809493 19671655

[109] Beaudoin C Jean S Bessette L Ste-Marie LG Moore L Brown JP Denosumab compared to other treatments to prevent or treat osteoporosis in individuals at risk of fracture: a systematic review and meta-analysis. Osteoporos Int. (2016) 27:2835–44. 10.1007/s00198-016-3607-6 27120345

[110] Kutilek S. denosumab treatment of severe disuse osteoporosis in a boy with spinal muscular atrophy. Acta Med Iran. (2017) 55:658–60. 29228533

[111] Kumaki D Nakamura Y Sakai N Kosho T Nakamura A Hirabayashi S. Efficacy of denosumab for glucocorticoid-induced osteoporosis in an adolescent patient with duchenne muscular dystrophy: a case report. JBJS Case Connect. (2018) 8:e22. 10.2106/JBJS.CC.17.00190 29642113

[112] Zhang D Potty A Vyas P Lane J. The role of recombinant PTH in human fracture healing: a systematic review. J Orthop Trauma. (2014) 28:57–62. 10.1097/BOT.0b013e31828e13fe 23454854

[113] Saraff V Högler W. ENDOCRINOLOGY AND ADOLESCENCE: osteoporosis in children: diagnosis and management. Eur J End. (2015) 173:R185–97. 10.1530/EJE-14-0865 26041077

[114] Catalano A Vita GL Russo M Vita G Lasco A Morabito N. Effects of teriparatide on bone mineral density and quality of life in Duchenne muscular dystrophy related osteoporosis: a case report. Osteoporos Int. (2016) 27:3655–9. 10.1007/s00198-016-3761-x 27589974

